# On the Oxalic Acid Diathesis

**Published:** 1844-06-29

**Authors:** H. Bence Jones


### RECORD OF MEDICAL SCIENCE.

### ON THE OXALIC ACID DIATHESIS.

By H. Bence Jones, M. A. &c.

The author commences his paper with a quotation from M. Vigla, who, in 1838, says, "Nous avions frequemment observeé dans les sedimente ou les cremors de l'urine de semblables cristaux (octaedriques) que leur forme aurait pu faire supposer être formés de chlorure de sodium, si la solubilité de ce sel, et la petite quantité qui s'en trouve dans l'urine, avaient permis de s'arrêter à cette idée.

Dr. Golding Bird, in 1842, stated that these octohedral crystals were oxalate of lime. No chemical proof having (as far as the author knows) been given, he was led to analyse the sediment. On examining urine for this purpose, the very frequent occurrence of these crystals in rheumatism was observed. In one case, in which the rheumatism was slight, the influence of diet and exercise on the mixed deposit of urate of ammonia and oxalate of lime was made the subject of experiment.

In other cases in which the crystals occurred, the symptoms were altogether different, irritation of the urinary organs being the most prominent. The concretion of the crystals into oxalate of lime gravel seemed (in one case at least) to be the cause of this diversity of symptoms.

The author observes that these crystals do not often occur in sufficient quantity to admit of analysis, but in Oct. 1843, he examined the urine of a patient of Mr. Cutler's, and at the same time three small renal calculi, which had been passed in July, August, and September. The urine under the microscope contained multitudes of octohedral mixed with some crystals of uric acid. All the calculi consisted of oxalate of lime mixed with uric acid.

The author has also examined cases of acute rheumatism, and always found the presence of these octohedral crystals in the urine of patients laboring under this disease. This deposit is also not unusually found mixed with urate of ammonia in chronic rheumatism. In one case he was enabled to make some experiments on the effects of diet and exercise on the deposit of urate of ammonia, and in it he observed that the octohedral crystals varied in quantity at different hours of the day. The daily results of this experiment are given very minutely, during the four weeks it lasted.

The author states it would be easy to multiply examples of the connection between octohedral crystals and rheumatism, but as it indicates no variation in the treatment of the disease, the fact seems only interesting as showing the close connection between the red deposit and octohedral crystals, and thus giving additional support to the theory of Professor Liebig regarding the origin of oxalate of lime.

The presence of octohedral crystals in the urine is frequently accompanied with symptoms of a totally different kind. The patient complains of pain in the loins, frequent desire to pass urine—which is sometimes small in quantity, at other times so large as to simulate diabetes. There are sudden calls to empty the bladder, and if they are delayed considerable pain is produced. The urine when examined contains only a slight cloud, which does not disappear on the application of heat. When examined by the microscope, this cloud is seen to consist sometimes entirely of octohedral crystals; more frequently of these crystals mixed with globules of mucus, and sometimes there are large and small scales of epithelium.

The symptoms closely resemble those produced by a small calculus in the kidney; and in one case they suddenly ceased after sharp pain in the course of the right ureter, and slight retraction of the testicle.

The author concludes by observing that the treatment which has proved most beneficial has been that which improved the general health. In two of Mr. Cutler's cases the symptoms appeared to follow mental anxiety. Medicines had little effect, but as the cause for anxiety disappeared, the symptoms ceased.—*Trans. Royal Med. and Chir. Society.*

